# Patient involvement in rheumatology outpatient service design and delivery: a case study

**DOI:** 10.1111/hex.12478

**Published:** 2016-06-27

**Authors:** Savia de Souza, James Galloway, Carol Simpson, Radka Chura, Joanne Dobson, Nicola J. Gullick, Sophia Steer, Heidi Lempp

**Affiliations:** ^1^ Academic RheumatologyKing's College LondonLondonUK; ^2^ RheumatologyKing's College Hospital NHS Foundation TrustLondonUK

**Keywords:** outpatient services, patient education, patient involvement, rheumatology, service delivery, service user

## Abstract

**Background:**

Patient involvement is increasingly recognized as important within the UK National Health Service to ensure that services delivered are relevant to users’ needs. Organizations are encouraged to work with service users to achieve excellence in care. Patient education can improve health outcomes and reduce health‐care costs. Mobile technologies could play a vital role in this.

**Aim:**

Patient‐centred development of innovative strategies to improve the experience of rheumatology outpatients.

**Case study:**

The Group Rheumatology Initiative Involving Patients (GRIIP) project was set up in 2013 as a joint venture between patients, clinicians, academics and management at a London hospital. The project saw (i) the formation of an independent patient group which provided suggestions for service improvement – outcomes included clearer signs in the outpatient waiting area, extended phlebotomy opening hours and better access to podiatry; (ii) a rolling patient educational evening programme initiated in 2014 with topics chosen by patient experts – feedback has been positive and attendance continues to grow; and (iii) a mobile application (app) co‐designed with patients launched in 2015 which provides relevant information for outpatient clinic attendees and data capture for clinicians – downloads have steadily increased as users adopt this new technology.

**Conclusion:**

Patients can effectively contribute to service improvement provided they are supported, respected as equals, and the organization is willing to undergo a cultural change.

## Background

Patient and public involvement (PPI) in health‐care planning, service development, healthcare policy and research has gained increased importance over the past two decades.^1^, ^2^ In research, funding bodies are now commonly requesting demonstration of PPI in applications, with lay involvement viewed by some as ethically mandatory.^2^, ^3^ The potential benefits of PPI in research (such as better quality results, more relevant research for patients and better translation into clinical practice) are increasingly acknowledged.^2^, ^3^ Guidance and examples of how to involve patients and the public in research have been published.^4^, ^5^, ^6^ In Academic Rheumatology at King's College London, we have been involving patients (as ‘patient experts’) for over a decade in teaching, research and co‐authoring publications.^7^, ^8^, ^9^, ^10^, ^11^, ^12^, ^13^, ^14^, ^15^, ^16^, ^17^

In clinical practice, the traditional paternalistic attitude of ‘Doctor knows best’ had been the *status quo* for many years and quality of care equated to good clinical care. The latter has since changed to include dimensions such as safety, clinical effectiveness and patient‐centredness, and is now embedded in the UK National Health Service (NHS) constitution.^18^, ^19^ As users and funders of the NHS, patients are stakeholders and entitled to influence the way it is run.^20^ Leading hospitals in the field of patient‐centred care have patients involved in a range of formal quality functions, such as sitting on the hospital board's quality committee.^21^

Since 2001, the UK Department of Health (DoH) has encouraged reforms to transform the relationship between healthcare professionals and patients into a partnership. Patients are encouraged to take more control of their own health and be involved in health service development.^22^, ^23^, ^24^ The DoH promotes PPI in service planning, design, development and delivery to improve services and better patient outcomes.^25^ Individuals with long‐term conditions become experts in their condition through lived experience. This valuable experiential knowledge can contribute to the improvement of healthcare delivery.^26^, ^27^, ^28^ Involved patients can act as ‘knowledge brokers’ by exchanging knowledge and building links between service users and providers.^29^ User involvement has been successfully adopted worldwide in mental health, HIV and cancer care.^30^, ^31^, ^32^, ^33^, ^34^, ^35^ At Bristol Royal Infirmary (UK), patients have been involved with the redesign of rheumatoid arthritis (RA) outpatient services and continue to attend review meetings.^36^, ^37^

In 2010, the Council of the European Union emphasized that healthcare needs to become more patient‐centred and involve patients, particularly those with chronic illness.^38^ Patients with rheumatic diseases need to attend their general medical practice and hospital on a continual basis. Currently, patients with RA report a lack of support following diagnosis, and that the information provided to them is insufficient.^39^ Education can empower patients to self‐manage their condition and in RA has been shown to reduce disease activity in the long‐term.^40^ Patient education can also help reduce the administrative burden for healthcare professionals and ultimately lead to less use of services and a substantial cost‐saving to the NHS.^41^

In the UK, digital technologies are commonly used across all social groups below the age of 60.^41^ In 2015, 71% of citizens owned a smartphone and 49% owned a tablet.^42^ Yet, only 2% of the population reported a digitally‐enabled transaction with the NHS in 2014.^41^ Better integration and more widespread use of technology within the healthcare system is now considered a top priority by the UK Government. The DoH recognizes that a framework for this needs to involve patients, the public, healthcare providers, researchers and suppliers.^41^ Mobile technology could be a powerful tool in patient self‐management and be used to collect screening data for the hospital, for example the Health Assessment Questionnaire (HAQ) to assess functional disability. Mobile applications empower self‐care and can improve patient outcomes.^41^

Against this background of the UK policy of patient involvement in research, service planning and delivery; rheumatology clinicians at King's College Hospital expressed an interest in establishing a patient group to help with organizational difficulties and suggest improvements to advance rheumatology outpatient services. Staff and patient experts suggested it was important that the patient group remained independent so as not to be influenced or constrained by clinicians and hospital management. Therefore in 2013, the clinical and academic teams, along with patient experts, applied jointly for a year's funding proposing a new initiative to extend patient involvement into rheumatology outpatient services at King's College Hospital NHS Foundation Trust.

### Aim and objectives

The aim focused on a patient‐centred development of innovative strategies to improve the patient experience of rheumatology outpatient services, with three distinctive strands: (i) formation of an independent patient group (IPG), (ii) initiation of patient educational evenings, and (iii) development of a mobile application.

## Case study

The Group Rheumatology Initiative Involving Patients (GRIIP) project proposal, named by one of our patient experts, received an Innovation Award from the Health Innovation Network South London in 2013. Ten project meetings were held between November 2013 and October 2014. These were attended by patient experts, clinicians, academics and hospital management with agendas set jointly by patients and the project lead. Meetings were chaired by a patient expert with ‘action points’ set, including who was responsible for these to be dealt with, and followed up at subsequent meetings. Project meeting minutes were circulated amongst the GRIIP team.

### Independent patient group

#### Process

The IPG was set up in January 2014 to improve and drive change in rheumatology outpatient services. It was decided by the GRIIP team that recruited patients should be as representative as possible of our rheumatology outpatient population. Ten patients (eight females : two males) were recruited by clinic nurses with an age range of 29–67 years and of diverse ethnic backgrounds (three White British, two White European, three Black Caribbean, two Asian). Group members lived with the following long‐term conditions: systemic lupus erythematosus (3), RA (2), ankylosing spondylitis (2), psoriatic arthritis (1), polymyositis (1) and mixed connective tissue disease (1).

The project lead and patient experts drew up ‘Terms of reference’ for the group, which included confidentiality (see Supporting information), and these were agreed to by patient group members. Ten monthly meetings were held early evening in the Academic Rheumatology Department between January and October 2014. Agendas were drawn up by the co‐chairs (departmental patient experts) based on clinic observation and personal experience. Sample agenda items were experiences of blood tests, the rheumatology outpatient waiting room and the appointments system (see Supporting information for full list). IPG members completed evaluation forms every 3 months and were reimbursed expenses.

Minutes from the meetings were sent to clinic staff and management, after approval from a patient chair. Based on feedback from these minutes, a list of active issues was drawn up in October 2014 by the rheumatology clinical lead listing the issue, action to be taken, when the task is be completed by and who is responsible (see Supporting information).

#### Outcomes

Mean attendance at IPG meetings was 62%. Evaluation showed they were well received (mean rating 7.5/10) with patients finding them informative and useful to meet other patients, share ideas/experiences, hear different views, learn about rheumatology clinic initiatives and current/future research. Some patients provided the following formal feedback:

A good group for discussion.

Happy with how the meetings have been done.

Stick to the agenda – sometimes points are drifted from.

I wanted to give something back.

Successful service improvements are listed in Table 1. Two active issues remain outstanding as long‐term goals: (i) dedicated musculoskeletal training of general practitioners, and (ii) the purchase of bespoke and comfortable seating for the waiting room suitable for patients with musculoskeletal conditions, for example higher than average seat height with supporting arms.

**Table 1:** Successful outcomes from IPG feedback

Category	Outcomes
Outpatient waiting experience	Reduction in mean clinic wait times (67% of patients were seen within 30 mins of their appointment time in September 2014 compared with 47% in September 2013)
	Cleaner/tidier waiting area
	Clearer signs in the waiting area (see Supporting information)
	Two additional mobile tablets purchased so patients can fill out patient‐reported outcome measures (PROMs) whilst waiting for appointment (rheumatoid arthritis PROMs capture >70 per month since additional tablets purchased compared with approximately 30 per month previously)
Rheumatology clinical services	More helpful reception staff
	Friendlier Assessment Room nurses
	Patient consultations no longer interrupted by other staff
	All medical students now introduced to patient and verbal consent sought as to whether they can observe consultations
	Set up of a formal annual review process for patients
	Feet now examined as part of routine consultations
Allied health services	Longer opening hours for phlebotomy (7.30 am–5.45 pm, previously closed circa 3 pm)
	A review of physiotherapy services provision for rheumatology patients
	Better access to allied health services, for example podiatry
	More timely delivery of home medication (92% of patients received their medication on time in October 2014 compared with 48% in June 2014)
	Launch of the Local Care Record to securely share patient information between the hospital and local GP surgeries^43^

IPG, Independent Patient Group.

A big success of the IPG was a meeting held at the hospital in July 2014 with the Clinical Director of a large‐scale, national home medication delivery company. This was specially arranged by a co‐chair of the patient group in response to patient complaints of delayed/failed deliveries and unreasonable call wait times on premium rate phone numbers when contacting the company.

Patients who attended the meeting, along with representatives from the rheumatology outpatient clinic and hospital pharmacy, received a detailed explanation about the company's logistical challenges and what steps they were taking to remedy the situation. A productive question and answer session followed, which gave patients an opportunity to express how delays in receiving their medication had personally affected them and to vent their frustration. The Clinical Director responded that it was important to hear patients' experiences first‐hand and apologized for the company's recent failings. The outcome of the joint meeting was reported back to all clinic staff at the subsequent clinical governance meeting.

### Patient educational evenings

#### Process

Patient educational evenings were planned to provide information and support to rheumatology outpatients. The sessions lasted 1.5 h, ran early evening approximately every 6 weeks in Spring/Summer 2014–2015 (due to patient preference) and were held in the hospital boardroom with refreshments provided. Patients were invited through advertisement in the rheumatology outpatient department (posters and flyers) and/or personally by clinicians. Carers/family members were also welcome to attend.

Topics for the evenings were patient‐initiated and decided in advance between GRIIP team members. Titles for talks were discussed between speakers and patient experts to make them ‘patient‐friendly’. Each evening had 1–2 speakers who were rheumatology clinicians or allied healthcare professionals. It was of importance to include members of the wider multidisciplinary team (e.g. physiotherapists, occupational therapists, podiatrists and psychologists) as rheumatic diseases can be complex and often require management input from many health disciplines. See Table 2. for a list of talk topics and Supporting information for a sample poster/flyer.

**Table 2:** Patient educational evening talks 2014–2015

Date	Talk title
Apr 2014	Adjusting to a new diagnosis for patients with musculoskeletal conditions
June 2014	Using exercise and adaptations to best manage your musculoskeletal condition
Sept 2014	Protecting yourself against infections
Apr 2015	Understanding inflammatory arthritis
	Oral health in rheumatoid arthritis
June 2015	Foot health in inflammatory arthritis
	Management of and practical advice about ‘flare ups’
Aug 2015	Understanding anti‐rheumatic drugs
	How to make the most out of your pharmacist

After each talk, patients had the opportunity to share experiences and pose questions to the speakers, a rheumatology consultant, clinical nurse specialist and patient experts. Patients who attended in 2014 were asked to complete an evaluation form (see Supporting information) after each session.

#### Outcomes

Mean attendance at patient educational evenings in 2014 was very low initially (mean five patients/carers per meeting). However, they were extremely well received by those who attended (mean rating 9.4/10). With increased publicity, attendance improved in 2015 and 22 patients/carers attended the latest meeting in July 2015.

Patients found the evenings informative, helped them with ‘learning to cope’, and some wished they could have attended meetings like this when they were first diagnosed. The following are some patient accounts from the evaluation forms:

Provided with facts.

Use of plain language to make explanations easier.

Newly diagnosed and unsure what to expect. The evening was an eye‐opener and the talks were very much enjoyed.

Everybody relating to and discussing to try and identify experiences of the condition.

Suggestions given were to improve advertisement/publicity of future meetings. Informal feedback to patient experts from patients attending was that they found it very helpful to have other patients to talk to, who could understand what they were going through and give advice on how to self‐manage. Newly diagnosed patients in particular welcomed seeing patients who lived with RA for a long time who were mobile, which instilled hope. The patient educational evenings are to continue in Summer 2016.

### Mobile application

#### Process

Two patient focus groups were held with IPG members in January 2014 to discuss development of the mobile application (app) and proposed content. Patients were given the opportunity to express their wants and needs, and this ‘wish list’ was taken by a senior clinician to a local app developer. A high priority for patients was confidentiality and security of their information. Alongside the patients' requirements, clinicians also wanted key patient‐reported outcome measures, such as the HAQ, captured.

The app developer was chosen because they had previously developed a successful app for another NHS Trust. Several meetings took place between them and a senior clinician to bring the rheumatology department app into fruition. A prototype of the app was presented to the IPG in August 2014 by its developers for further feedback on design/content and ease of use, before being refined ahead of its launch.

The rheumatology department app was officially launched during a patient educational evening in April 2015 and was advertised to patients via a poster in the outpatient clinic, on patient appointment letters and through word‐of‐mouth. Feedback was welcomed from patients if there were any errors within the app or difficulties encountered with downloading it. In January 2016, download statistics were obtained from the developer and patients were invited to provide feedback.

#### Outcomes

See Table 3. for mobile app features. The app had been downloaded by 190 users by January 2016 on both Android and iOS operating system devices. Patients' comments about the app were as follows:

**Table 3:** Key components of the mobile application

Category	Features
General hospital information	Map of hospital buildings
	Location and opening hours of departments (e.g. rheumatology, radiology, physiotherapy, phlebotomy and pharmacy)
	Parking charges
	Location of local cash machines and cafeterias
Rheumatology clinic information	Names and positions of clinic staff
	Useful contact numbers, for example reception desk, appointment line, consultants’ secretaries, emergency nurse‐run helpline
Patient information	Links to external web sources of reliable patient information such as Arthritis Research UK, National Rheumatoid Arthritis Society, National Ankylosing Spondylitis Society, Lupus UK and Myositis UK
Miscellaneous	Patients can enter their own appointment information (e.g. clinic dates, when blood tests due), which then integrates with the calendar function on their mobile device
	Patients can take and store a picture of their blood test form to show on their mobile device screen when they go for their blood test, which negates the need to carry the original paper form
	Patients can complete and securely submit a HAQ to be uploaded to their patient record ahead of their clinic appointment

HAQ, Health Assessment Questionnaire.

I like the app because it's so handy to have all the info I need about my arthritis in one place: whether it's when is my next blood test or hospital appointment, phone numbers for my drug delivery or where to get a nice cup of coffee.

I'm not quite sure of the purpose of it, now it feels like an extra calendar and phone contact app. Will there be a connection to test results, etc.?

I found it very informative and would like to be able to access blood results as well in the near future. It was quite easy to access the information and I will make sure to use it often.

## Discussion

### Independent patient group

The IPG has raised the profile of the patients' voice to the rheumatology outpatient clinic and helped instigate changes and improvements over a period of 12 months and beyond. The process allowed patients to directly contribute to shaping the services they receive long‐term and to realize their opinions were of value to clinic staff and hospital management. One of the unintended consequences of patient group formation was that it helped to establish an informal support network for patients with others who had shared a similar experience of living with a long‐term musculoskeletal condition. Similar reasons for patient engagement were given in a 3‐year NHS service improvement programme for stroke services in two London Boroughs.^28^

Since the GRIIP project ended in October 2014, the IPG operates on an *ad hoc* basis with members being contacted via email, by a departmental patient expert, as and when opinions are required on clinic proposals. Members are also welcome to continue to report any unsatisfactory clinic issues they experience or observe. These will be added to the active issues identified list. Initially, the feedback mechanism was more formal, but now that relationships have been established between patient experts, clinicians and management; patient experts can informally and directly raise clinic issues with the clinical team and/or hospital management at any time.

Although we tried to include a diverse range of members for the patient group, recruitment was somewhat opportunistic and how representative they are of the wider patient population can be open to question.^44^ Also, the two patient expert co‐chairs have been involved in research within the academic rheumatology department for many years. Patients who interact with clinicians and other project team members need to have certain qualities, such as a strong character and confidence, and can therefore be atypical of their peers.^45^ However, the extent to which involved patients influence service design and improvement seems to be of greater importance than their perceived ‘representativeness’.^45^

### Patient educational evenings

Patients who attended the educational evenings relished the chance to discuss informally any concerns they had about their condition with patient experts and the clinical team. It is difficult to assess whether patient educational evenings have resulted in better self‐management and lower unplanned outpatient attendances at this point, but written and verbal feedback from patients has been positive.

### Mobile application

Current widely‐available rheumatology patient apps are for symptom tracking, lifestyle monitoring or act as medication reminders.^46^ They tend to not be viewed as useful by rheumatologists due to a lack of relevant data capture, for example the HAQ.^46^ Our app is information‐specific for our own hospital and rheumatology department, includes the HAQ and is anticipated to reduce patient non‐attendance. We plan to carry out an audit in 2016 to assess whether non‐attendance rates have lowered as a result. King's College Hospital NHS Foundation Trust will use the rheumatology mobile app as a pilot for future wider use of digital communications to aid patient care at the hospital; for example, the gastroenterology department now has a similar app for their patients. Our app has also now gained international recognition, having recently won an award for mobile application design in healthcare.^47^

### Challenges and issues

There were many delays in getting the project off the ground due to administrative and financial infrastructure barriers across both institutions, for example the set‐up of honorary contracts at the hospital for patient experts and reimbursement of expense payments. Ongoing support from hospital management was lacking as was attendance/punctuality at GRIIP project meetings from clinicians and management, due to other pressing commitments. This resulted in IPG members feeling frustrated as little regular feedback from clinicians and hospital management was received.

Once the GRIIP project ended in October 2014, it was planned that the two patient experts/IPG co‐chairs would attend King's College Hospital NHS Foundation Trust rheumatology department business meetings to continue addressing any outstanding active issues. However, after a trial period, it was agreed by all parties concerned that business meetings were not the best forum and a separate small group was set up between a patient expert, a consultant, a clinical nurse specialist and the rheumatology outpatient services manager to continue with this work.

Barriers to successful implementation of PPI have been extensively published. These include the time commitment and expertise it takes to make PPI work, the belief by clinicians and managers that patients cannot make an effective contribution, and the perceived threat to organizations of ‘losing face’ by sharing their organizational shortcomings and difficulties with their service users.^28^, ^48^, ^49^, ^50^, ^51^, ^52^ Indeed, a lack of organizational supportive infrastructure; scepticism or resistance to act on data by clinicians/health‐care managers; and/or uncertainty over what would be an effective intervention can hinder implementation of patient feedback.^27^, ^53^

There is a real danger of patient involvement being sidelined when patients are expected to integrate into pre‐existing organizational structures such as management meetings, whereby their ability to effectively contribute can be hampered by a lack of familiarity with the system and power differentials.^54^ This is when patient involvement runs the risk of becoming a ‘rubber stamping’ exercise. Therefore, it is imperative for patients involved to engage as equals with healthcare professionals in meetings, be able to challenge them and believe they can make a valuable contribution.^45^ It can take months or years for PPI processes to fully develop and become embedded in organizations, and a further time lag before PPI leads to quality improvement and observable changes.^28^, ^55^

### Future plans

To build on the GRIIP project, we plan to (i) continue the patient educational evenings in Summer 2016, (ii) update the rheumatology information pages on the hospital website, (iii) run a monthly patient expert‐led clinic for newly diagnosed patients (in parallel with a rheumatology consultant clinic) where patients can seek advice and support from someone who has lived with and successfully managed a rheumatic disease for a long time, and (iv) expand the capabilities of the mobile app so it can link into the electronic patient record (thereby enabling patients to view their blood results, make/change appointments and view their medical notes).

## Conclusion

Patients are interested in helping shape the services they receive, and have played an important and active role to drive change in outpatient services and improve patient education/self‐management. Patient involvement can only really work if all parties are engaged in the process. It requires time and commitment to establish an effective partnership. Clinicians can sometimes perceive patient involvement as a threat to their knowledge and expertise; however, patients have a separate complementary role and can provide fresh perspective on existing problems. Teamwork is important, and mutual respect is key to this joint enterprise. The way forward is for greater cooperation between service providers and service users.

## Source of funding

Health Innovation Network South London, London, UK.

## Conflicts of interest

None.

## Supporting information


**Appendix S1.** Active issues identified by the IPG 27.10.14.

**Appendix S2.** Evaluation form for patient educational evening June 2014.

**Appendix S3.** IPG meeting topics.

**Appendix S4.** King's College Hospital rheumatology app screenshots.

**Appendix S5.** New signs in outpatient department.

**Appendix S6.** Poster/flyer for patient educational evening July 2015.

**Appendix S7.** Terms of Reference for the IPG.
