# Supplementary material for: Patient involvement in rheumatology outpatient service design and delivery: a case study
Source: Health Expect. 2016 Jun 27;20(3):508–18. doi: 10.1111/hex.12478 (PMC5433532; doi:10.1111/hex.12478)
Supplement: Supplementary file 1 — Appendix S1. Active issues identified by the IPG 27.10.14. [file HEX-20-508-s001.pdf]

Active Issues identified by Independent Patient Group / Patient representatives – 27.10.14

Categories	Active Issues	Actions taken	Responsible	To be completed (date)
**Suite 3 issues**	• Untidiness<br>• Cleanliness	[redacted] and [redacted]. informed about first two issues. [redacted] and [redacted] reported that untidiness and cleanliness have improved. Further ad hoc monitoring necessary.	[redacted] to continue to liaise with [redacted] / Suite 3 team as necessary depending on findings	Ongoing
	• Reception staff	Ongoing unresolved issue, although slight improvement. [redacted], Clinic staff and patients are aware of the unsatisfactory situation. [redacted] asked for understanding of the pressure reception staff are under too, however attitudinal problems towards patients still observed.	Rachael to continue to liaise with [redacted]/Suite 3 team. Patients to give specific examples of unsatisfactory interactions if possible	Ongoing
	• IMPARTS	Other specialities have now come on board with IMPARTS too, which may generate momentum. Meeting with all the departments arranged with [redacted], who is in charge of IMPARTS.	[redacted]	Ongoing
	• Chairs	Was revisited with [redacted]. Trial chairs to be installed shortly.	[redacted]	Ongoing
	• Waiting times	Have improved in general; however individual consultants' waiting time shown on board much preferred. To be further discussed with Rachael W. and Alex F.	[redacted]	Ongoing

	• Nursing staff	Nursing staff in Assessment Room - situation has improved in relation to attitude towards patients.	[redacted]	Ongoing monitoring
[redacted]	• Failed deliveries, interruption of patients treatment	Situation has improved, review meeting with KCH clinicians in late September. Patients need to inform clinicians in the clinic when delivery is not satisfactory. Info also through the IPG minutes.	[redacted] (re IPG)	Ongoing
**Appointments issues**	• Cancellations Rescheduled appointment delayed<br>• Multiple cancellations<br>• Short-notice cancellations<br>• Wrong clinics booked at follow-up	[redacted] is doing lots of work around these issues, situation has generally improved.<br>Appointment issues are reviewed regularly during departmental business meetings, and number of multiple appointment cancellations has gone down from two to one figure last time this issue was reviewed. Staff have to give 8 weeks' notice if they are on holiday, but some events are unpredictable, e.g. illness, crisis at home etc.	[redacted]	Ongoing
**Consultation interruptions**	• Interruptions by colleagues<br><br>• Information / choice about medical students	This issue has been discussed in the department, and all clinicians are aware that patients find the consultation interruptions unsatisfactory and are trying to keep these to a minimum.<br>All staff reminded to offer patients choice of whether students are present during their consultation. If agreed INTRODUCE student.<br>More detailed info necessary from patients in the IPG to find out about the impact the interruptions or medical students' presence have.	[redacted]	Discussed at IPG meeting (9/10) and not an issue with members present

**Communication from Hospital to GP and patient**	• Hospital / GP – letters going to wrong surgery<br>• Hospital / patient – timeliness of appointment letters	Specific examples (evidence) needed by patients, so that clinicians can act upon this via their admin staff.<br><br>Letters re: appointments sent second class and sometimes don't arrive until date of appointment has passed. Contributed to by reception staff sometimes not making appointment in Suite 3. Less of a problem for patients who have supplied mobile telephone number – text reminders useful. [redacted] suggested patients also need to be more pro-active e.g., phone clinic for appointment if reception unable to do so at time of current appointment/have reminders on phone, calendar.	Patients<br><br>Patients	Ongoing<br><br>Ongoing
**GP skills / knowledge**	• Patients acting as conduit of advice between hospital and GP	The clinical team is aware that closer collaboration and updating of MSK training of GPs is necessary. This issue will be addressed in the future, not immediately	[redacted]	Review in January 2015.
**Therapies referrals**	• Lack of consistency<br>• Annual review process	Consistency could be improved by flagging patients requiring annual review and a proforma of all domains of the annual review can be developed in the future. Issue raised in staff meeting and all clinicians aware.<br>Meanwhile suggest patients be pro-active and let clinicians know when and what referral they want and not wait for staff to ask if necessary.<br>Feedback regarding quality of physiotherapy service sent to physiotherapy lead and initiatives to improve this under way.	[redacted]	Review January 2015

**Patient Evenings**	Restart Spring 2015	Clinic to be more proactive around advertising the patient evenings – nurses, doctors, consultants, posters, sign on reception desk, email, etc.<br><br>[redacted] to recruit Registrars to give talks and take responsibility for ensuring the information reaches as many patients as possible	[redacted]	March 2015 – September 2015
**Patient App**	In production	Meeting arranged for 10/11/14 with IPG members for them to comment on progress so far and make suggestions	[redacted] ([redacted] re: contract and final Report and Finances to AHSN)	December 2014
