# Supplementary material for: Patient involvement in rheumatology outpatient service design and delivery: a case study
Source: Health Expect. 2016 Jun 27;20(3):508–18. doi: 10.1111/hex.12478 (PMC5433532; doi:10.1111/hex.12478)
Supplement: Supplementary file 2 — Appendix S2. Evaluation form for patient educational evening June 2014. [file HEX-20-508-s002.docx]

**King’s 2nd Patient Educational Evening 24.06.14**

EVALUATION SHEET

Please indicate on the scale, by circling one number, your opinion of the ***value*** of this meeting?

I..........I……….I……….I……….I……….I……….I……….I……….I……….I……….I

0 1 2 3 4 5 6 7 8 9 10

Little value Great value

What did you find particularly ***useful*** about the meeting?

……………………………………………………………………………………………………………………………………………………….

……………………………………………………………………………………………………………………………………………………….

……………………………………………………………………………………………………………………………………………………….

How could the meeting be ***improved***?

……………………………………………………………………………………………………………………………………………………….

……………………………………………………………………………………………………………………………………………………….

……………………………………………………………………………………………………………………………………………………….

Are you here this evening as a patient/carer/clinician/researcher/other (please specify)?

………………………………………………………………………………………………………………………………………………………..

Please indicate on the scale by circling one number, how you rate the ***room/catering*** for the meeting?

I..........I……….I……….I……….I……….I……….I……….I……….I……….I……….I

0 1 2 3 4 5 6 7 8 9 10

Unsatisfactory Satisfactory

Finally, how did you hear about the meeting?

……………………………………………………………………………………………………………………………………………………….

Thank you for your comments
