# Supplementary material for: Patient involvement in rheumatology outpatient service design and delivery: a case study
Source: Health Expect. 2016 Jun 27;20(3):508–18. doi: 10.1111/hex.12478 (PMC5433532; doi:10.1111/hex.12478)
Supplement: Supplementary file 3 — Appendix S3. IPG meeting topics. [file HEX-20-508-s003.pdf]

IPG Meeting Topics

Jan 2014

- Any more thoughts on mobile app?
- What do you think newly-diagnosed patients would want out of patient educational evenings?
- Suggestions for improvement to the waiting experience in Suite 3.

Feb 2014

- Update on remuneration for participation in the IPG
- Issues in relation to prescribed medication
- Patient Educators for undergraduate medical students
- Feedback re: mobile app

Mar 2014

- Introduction of GRIIP project administrator
- Issues in relation to blood tests
- Opinions sought on developing a cardiac rehabilitation exercise programme for patients with musculoskeletal conditions
- Suggestions for patient educational evening topics

Apr 2014

- Presentation on My Health Locker

May 2014

- Issues relating to home medication delivery
- Issues in relation to appointments
- 3rd Patient education evening topic (September 2014): vaccinations
- Communication between hospital and GPs

June 2014

- Department research update by Dr Galloway

July 2014

- Open meeting with home medication delivery company and King's College Hospital

August 2014

- Feedback on meeting with home medication delivery company
- Update on referrals to allied healthcare departments
- More suggestions for improving physiotherapy services
- Update on mobile app

- Information on next patient educational evening

September 2014

- Final update on GRIIP project work and all issues raised at IPG meetings

October 2014

- Preview of mobile app prototype
- Discussion on future of the IPG
