# Supplementary material for: Patient involvement in rheumatology outpatient service design and delivery: a case study
Source: Health Expect. 2016 Jun 27;20(3):508–18. doi: 10.1111/hex.12478 (PMC5433532; doi:10.1111/hex.12478)
Supplement: Supplementary file 4 — Appendix S4. King's College Hospital rheumatology app screenshots. [file HEX-20-508-s004.pdf]

King’s College Hospital Rheumatology App Screenshots

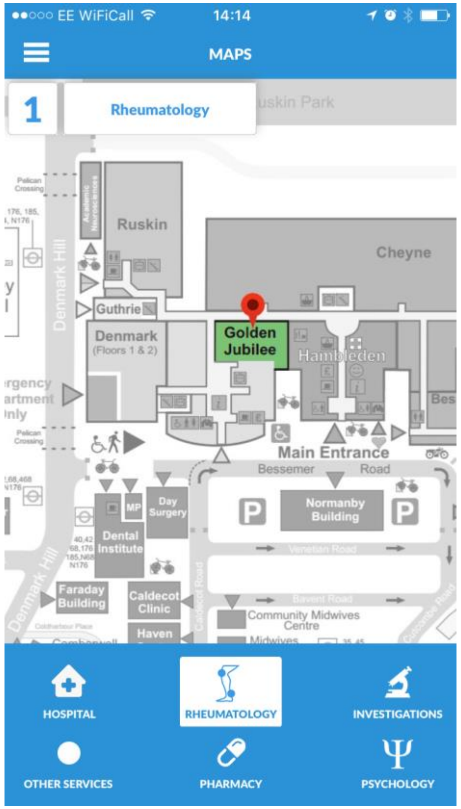

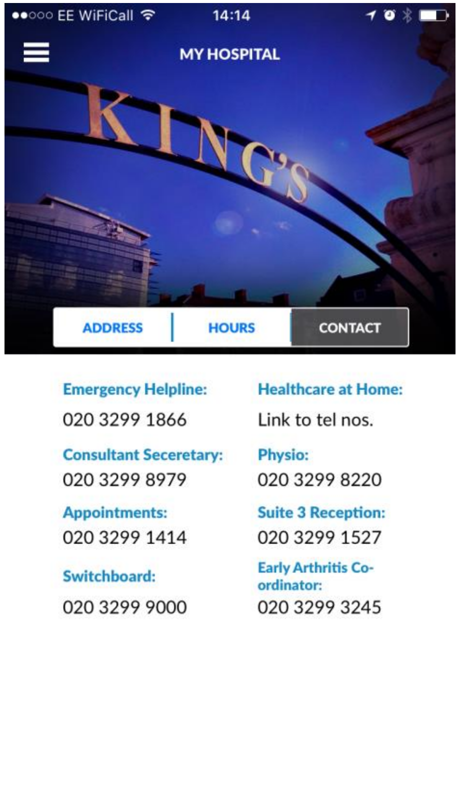

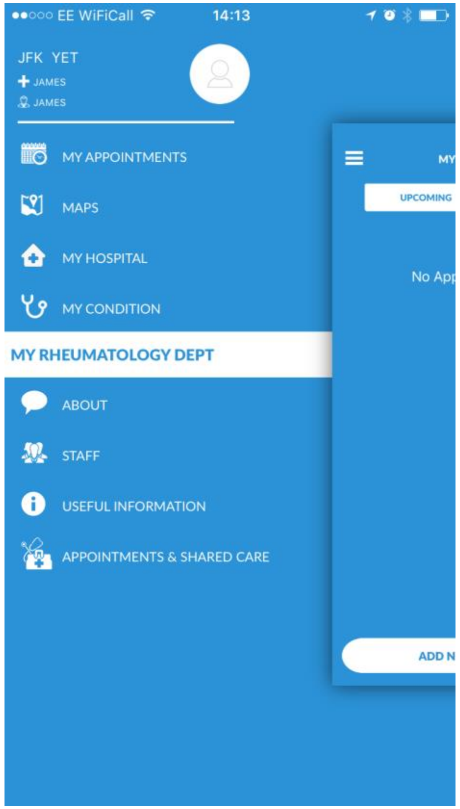

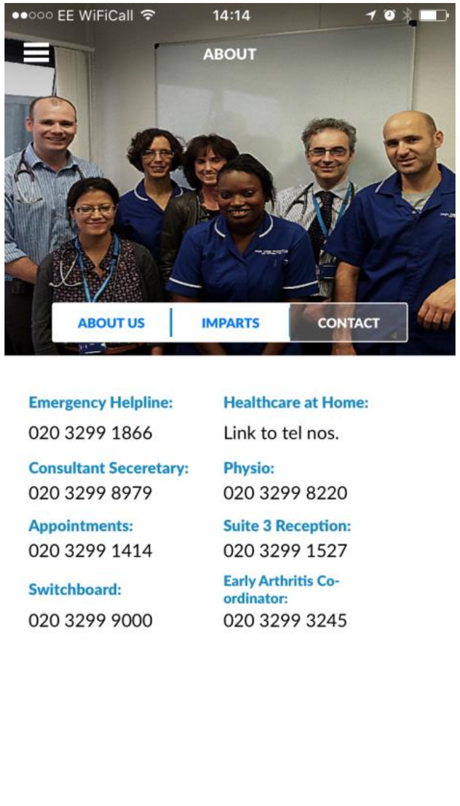

Images courtesy of Ampersand Mobile

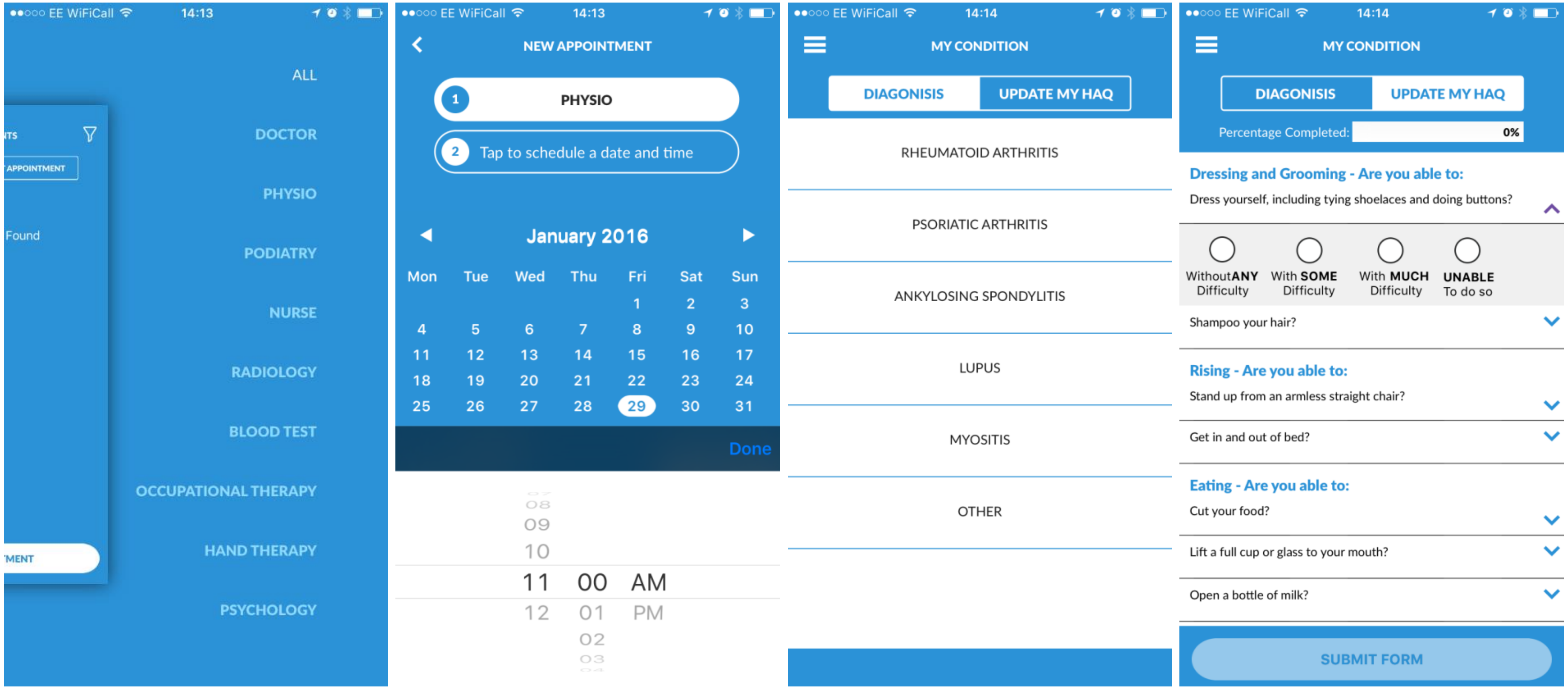

Images courtesy of Ampersand Mobile
