# Supplementary figures and images for: Patient involvement in rheumatology outpatient service design and delivery: a case study
Source: Health Expect. 2016 Jun 27;20(3):508–18. doi: 10.1111/hex.12478 (PMC5433532; doi:10.1111/hex.12478)

## New Signs in Outpatient Department

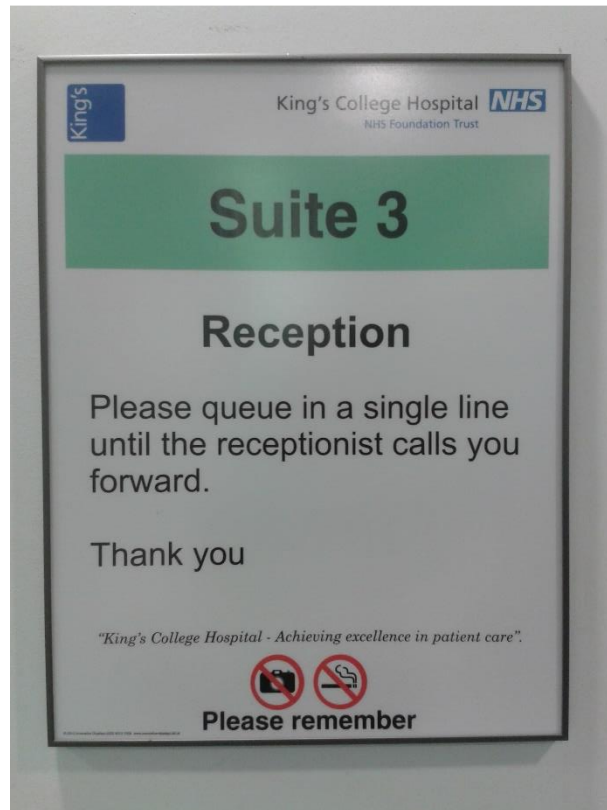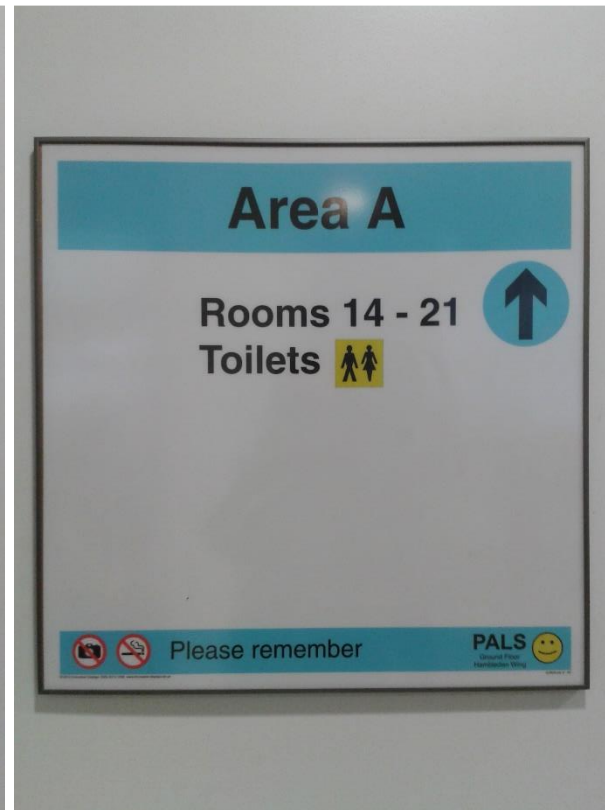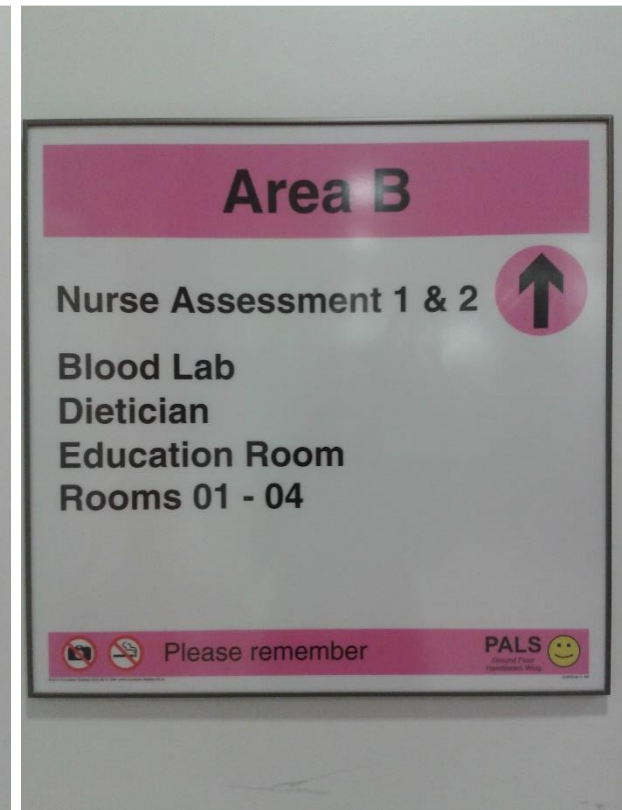

Supplement: Supplementary file 5 — Appendix S5. New signs in outpatient department. [file HEX-20-508-s005.pdf]
