# Supplementary material for: Patient involvement in rheumatology outpatient service design and delivery: a case study
Source: Health Expect. 2016 Jun 27;20(3):508–18. doi: 10.1111/hex.12478 (PMC5433532; doi:10.1111/hex.12478)
Supplement: Supplementary file 6 — Appendix S6. Poster/flyer for patient educational evening July 2015. [file HEX-20-508-s006.pdf]

King's College Hospital NHS
NHS Foundation Trust

KING'S College LONDON

Patient Educational Evening for all patients attending the Rheumatology Clinic at King's

Wednesday, 29th of July 2015, 5.30pm-7pm

Everybody is very

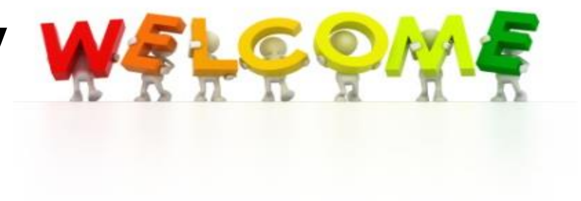

1)'Understanding anti-rheumatic drugs' by Dr. Andy Jeffries
2) 'Making the most of your pharmacist' by Nicola Torrens

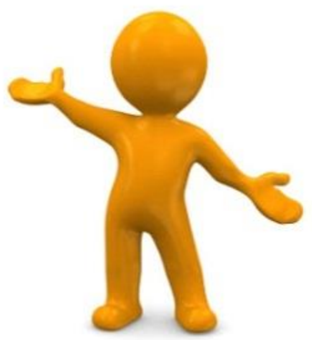

Light refreshments will be provided.

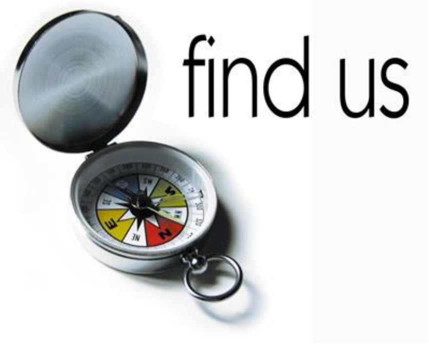

at Boardroom, Hambleden Wing
King's College Hospital, (will be signposted)

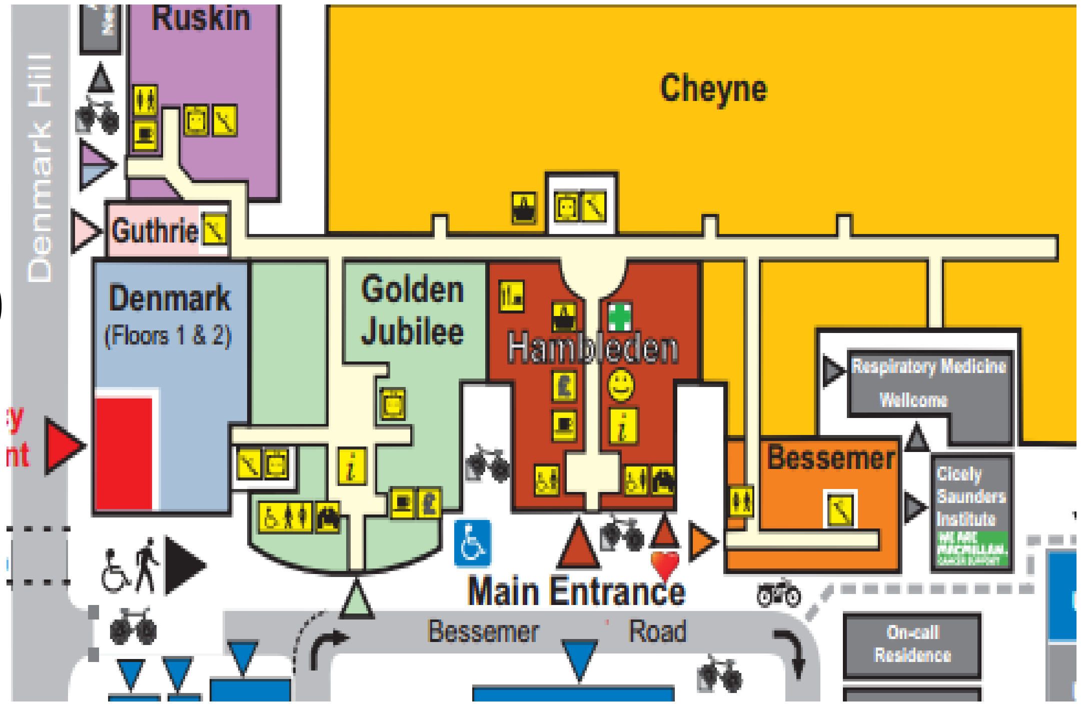

Any questions please call 0207 848 5604 or 0208 480 8799.
