# Supplementary material for: Patient involvement in rheumatology outpatient service design and delivery: a case study
Source: Health Expect. 2016 Jun 27;20(3):508–18. doi: 10.1111/hex.12478 (PMC5433532; doi:10.1111/hex.12478)
Supplement: Supplementary file 7 — Appendix S7. Terms of Reference for the IPG. [file HEX-20-508-s007.pdf]

Terms of Reference for the Independent Patient Group (GRIIP)

To consider and agree:

- to *commit* to participating in the Independent Patient (IPG) group over 10 months (Janurary-October 2014) by attending 8-10 meetings
- to *keep* all information shared within the IPG *confidential*
- to *respond to* requests from the GRIIP Project Management group to achieve all 3 monthly Project Milestones within the set deadlines stated in the Academic Health Science Network application
- to *liaise* and *negotiate* with relevant members of the Project Management team (clinicians, academic, patients)
- to *monitor and provide* appropriate advice about how the requirements of the 12 months' Project and its findings might best be achieved and applied in the Department of Rheumatology
- to *work within* the given budget for the duration of GRIIP
- to *scrutinise and comment* on emerging data from the developmental work and relevant information gathered, e.g. evaluation, observations from a patient perspective to identify key findings and gaps that require raising awareness of and rectification within the Department of Rheumatology
- to *participate* in the dissemination of the findings from the work achieved, e.g. conference abstracts, newsletter contributions, publications
- to *contribute* to the written reports of progress that need to be submitted to the Academic Health Science Network.
